# Non-Local Mean Denoising Algorithm Based on Fractional Compact Finite Difference Scheme Effectively Reduces Speckle Noise in Optical Coherence Tomography Images

**DOI:** 10.3390/mi13122039

**Published:** 2022-11-22

**Authors:** Huaiguang Chen, Jing Gao

**Affiliations:** 1 School of Science, Shandong Jianzhu University, Jinan 250101, China; 2 Center for Engineering Computation and Software Development, Shandong Jianzhu University, Jinan 250101, China

**Keywords:** optical coherence tomography, speckle noise, image denoising, fractional compact finite difference scheme

## Abstract

Optical coherence tomography (OCT) is used in various fields such, as medical diagnosis and material inspection, as a non-invasive and high-resolution optical imaging modality. However, an OCT image is damaged by speckle noise during its generation, thus reducing the image quality. To address this problem, a non-local means (NLM) algorithm based on the fractional compact finite difference scheme (FCFDS) is proposed to remove the speckle noise in OCT images. FCFDS uses more local pixel information when compared to integer-order difference operators. The FCFDS operator is introduced into the NLM algorithm to construct a high-precision weight calculation so that the proposed algorithm can effectively reduce the speckle noise in the OCT images. Experiments on simulations and real OCT images show that the proposed method is comparable to other state-of-the-art despeckling methods and can substantially reduce noise and preserve image details such as edges and structures. Speckle noise removal can further promote the application of the proposed algorithm in medical diagnosis and industrial detection, as it has key research value.

Optical coherence tomography (OCT) is a non-invasive, high-precision imaging modality that is used in various fields, such as dermatology and ophthalmology [1]. However, as the OCT system uses the principle of light interference, the generated image is severely affected by speckle noise. This leads to a significantly low-quality image [2]. The layered structure and fine features of OCT images are blurred due to their low quality, which affects the accuracy of the diagnosis by the doctor [3]. An effective OCT image denoising algorithm can improve the image quality, thereby enhancing the quality of the diagnosis.

Recent researches show that either direct averaging [4] or hardware-based phase modulation [5] then averaging multiple OCT images can significantly suppress the speckle noise and enhance the OCT image quality with the cost of longer time data acquisition. However, algorithm-based speckle denoising methods are still in high demand. In addition, many despeckling algorithms have been proposed for OCT images, which are generally divided into two categories: Transform domain method and spatial domain method. Transform domain algorithms such as the wavelet shrinkage technique [6,7] and the curvelet shrinkage technique [8] usually reduce the speckle noise in OCT images by manipulating the size of the correlation coefficients. However, it is difficult to distinguish fine details from the speckle noise using the transform domain algorithms. Some artifacts may also be produced during the denoising process. Spatial domain algorithms are usually divided into local and non-local filters, such as partial differential equation (PDE) methods [9,10], non-local mean (NLM) methods [11,12,13], sparse representation (SR) methods [14,15,16,17], low-rank approximation methods [3,18,19,20], multi-frame dependent algorithms [21,22,23,24] and deep learning methods [25,26,27,28]. The deep learning method is a relatively advanced algorithm that requires a large amount of training data. The training of the deep learning models requires high-performance computing and is time-consuming. In addition, the estimated image obtained by the PDE algorithm is under-smooth, while the estimated image obtained by the SR, LRA, or multi-frame dependent algorithms are over-smooth. Hence, the NLM algorithm has attracted the attention of many scholars.

The NLM algorithm reconstructs image pixels by the weighted averaging of pixels in a predefined search window, where non-local weights are determined based on the similarity between different image patches rather than individual pixels. The NLM method, originally designed for Gaussian noise removal, has lately been applied to remove speckle noise from ultrasound images [29,30] and synthetic aperture radar images [31]. Furthermore, by designing a new weight calculation operator, some NLM-based filter filters are proposed for OCT image denoising. A double-precision NLM filter with a Gaussian anisotropic kernel function, instead of the traditional uniform kernel function, was previously proposed to effectively remove speckle noise [11]. Additionally, a two-stage NLM method using the uncorrupted probability of each pixel was introduced to suppress noise [12]. In literature [32], they have presented a novel despeckling technique for OCT, TNode, which is based on the non-local means algorithm with proper speckle statistics. This algorithm could remove the noise in the background, but it was not sufficient to remove the noise in a high-intensity region. In addition, an NLM method based on guided filtering was also proposed for the despeckling of the OCT images [13]. This algorithm could remove the noise in the background, but it was not sufficient to remove the noise in a high-intensity region [33]. An NLM method based on guided filtering was also proposed for the despeckling of the OCT images. However, these NLM-based methods could only marginally improve the performance of the despeckling system. The disadvantage of NLM methods is that they cannot accurately determine the weights using only the neighboring individual pixels. Hence, the structural similarity of noisy image blocks cannot be effectively represented. Based on existing research, it can be understood that further characterizing the topological structures as image edges and textures by substantially reducing noise and improving the robustness of the algorithm is a very critical scientific issue. To address this problem, we propose an improved NLM algorithm, which uses a fractional compact finite difference scheme (FCFDS) to accurately calculate the image similarity weight. The proposed algorithm can provide a better filtering effect, as it not only fully removes speckle noise but also effectively retains the topological structure of the image. The enhanced filtering effect can further promote the application of the OCT image data in medical diagnosis, industrial inspection, cultural relic restoration, and other fields. The improved despeckling algorithm has significant research value. The results of this study can provide important technical support for the early diagnosis and treatment of diseases. The primary aims of our research are:

(1) A template convolution operator based on the FCFDS is proposed herein. The FCFDS is a high-precision format, an extension of the compact difference format for integer-order equations. Generally, a format with higher precision implies the use of more points and a greater number of calculations. However, the FCFDS can achieve high accuracy with fewer points. Hence, using the FCFDS can reduce the computational complexity of the algorithm while maintaining its effectiveness.

(2) A bidirectional, high-precision image similarity weighting strategy based on the FCFDS is used. The information regarding the main structure of the OCT image is primarily concentrated in the horizontal and vertical directions. Therefore, to better preserve its structural information, we propose a bidirectional non-local weighting operator. In addition, we propose to use the high-precision FCFDS operator to construct the variance parameter in the vertical direction to improve the preservation of the detailed texture of the image.

(3) An OCT image denoising algorithm based on the FCFDS and NLM algorithm is proposed to remove noise while effectively preserving the structural information of the image.

The rest of the paper is organized as follows. Section 1 provides the detailed process of the proposed method. In Section 2, the simulation and real OCT images are studied, and the experimental results obtained are discussed. Section 3 concludes the paper.

## 1. Proposed Method

The main advantage of fractional differentiation over integer order differentiation is that fractional-order differential operators are non-local and provide an excellent tool for describing the memory and genetic characteristics of image processing. However, in the classic integer order model, the influence of fractional order differentiation is ignored [34]. In addition, the higher-order calculus derivatives and the generalized differential quadrature method can obtain higher-precision orders in the numerical solution of partial differential equations. These higher-precision orders are more conducive for applications in practical engineering calculation problems. However, the construction of higher-order derivatives or generalized differentials requires more mesh points, increasing the complexity of the algorithm and its computation Fractional differential operators have also achieved good filtering effects in image denoising, but they have fewer applications in OCT image denoising. In addition, the development of the FCFDS increases prospects for applications of the fractional derivative theory and improves the accuracy of calculations. This supports fractional calculus in the preservation of non-local image block detail information. The following two lemmas are used in the difference scheme to derive the FCFDS.

**Lemma** **1** ([35]). *Assume that $f\left(x\right)\in L_{1}\left(R\right)\cap {℘}^{\alpha +1}\left(R\right)$, and define the shifted Grünwald difference operator by*

(1) $$S_{\beta ,\gamma }^{\left(\alpha \right)}f\left(x\right)=\frac{1}{\beta^{\alpha }}\sum_{k=0}^{\infty }g_{k}^{\left(\alpha \right)}f\left(x-\left(k-\gamma \right)\beta \right)$$

*where α∈(0,1), β, γ, and k are constants; ${℘}^{\alpha +m}\left(R\right)=\left\{f\right|\int_{-\infty }^{+\infty }{\left(1+|\omega |\right)}^{\alpha +m}\left|\hat{f}\left(\omega \right)\right|d\omega <+\infty$, $\hat{f}$ is the Fourier transform of f}; gk(α)=(−1)kαk=Γ(k−α)Γ(−α)Γ(k+1). Then*

(2) $$S_{\beta ,\gamma }^{\left(\alpha \right)}f\left(x\right){=}_{-\infty }D_{x}^{\alpha }f\left(x\right)+o\left(\beta \right)$$

*uniformly for x∈R as γ⟶0, and o(β) represents the higher-order infinitesimal of β. ${}_{-\infty }D_{x}^{\alpha }f\left(x\right)$ is the Riemann–Liouville fractional derivative, which can be expressed as:*

$${}_{-\infty }D_{x}^{\alpha }f\left(x\right)=\frac{1}{\Gamma (n-\alpha )}\frac{d^{n}}{dt^{n}}\int_{0}^{x}\frac{f(x)}{{\left(t-\varepsilon \right)}^{\alpha +1-n}}d\varepsilon$$

*where ε is the integral variable, and n is the smallest positive integer greater than α.*

**Lemma** **2** ([36,37]). *$f\left(x\right)\in L_{1}\left(R\right)$, ${}_{-\infty }D_{x}^{\alpha +3}f\left(x\right)$ and its Fourier transform belong to $L_{1}\left(R\right)$. Defining the weighted and shifted Grünwald difference operator by*

(3) $$D_{\beta }^{\alpha }f\left(x\right)=\rho_{1}S_{\beta ,\gamma }^{\left(\alpha \right)}f\left(x\right)+\rho_{2}S_{\beta ,\gamma }^{\left(\alpha \right)}f\left(x\right)+\rho_{3}S_{\beta ,\gamma }^{\left(\alpha \right)}f\left(x\right)$$

*where $\rho_{1}=\frac{12pq-\left(6p+6q+1\right)\alpha +3\alpha^{2}}{12(pq-\gamma p-\gamma q+\beta^{2})}$, $\rho_{2}=\frac{12pq-\left(6p+6q+1\right)\alpha +3\alpha^{2}}{12(\gamma q-\gamma p-pq+p^{2})}$, $\rho_{3}=\frac{12pq-\left(6p+6q+1\right)\alpha +3\alpha^{2}}{12(\gamma p-\gamma q-pq+q^{2})}$, and γ, p, q are all integers. Then, we have*

(4) $$D_{\beta }^{\alpha }f\left(x\right){=}_{-\infty }D_{x}^{\alpha }f\left(x\right)+o\left(\beta^{3}\right)$$

*uniformly for x∈R as γ⟶0. Because of the symmetry of γ, p and q, it may be assumed that δ>p>q. By choosing γ=0, p=−1 and q=−2, we get*

(5) $$\rho_{1}=\frac{24+17\alpha +3\alpha^{2}}{24},\rho_{2}=-\frac{11\alpha +3\alpha^{2}}{24},\rho_{3}=\frac{5\alpha +3\alpha^{2}}{24}.$$

*According to Lemmas 1 and 2, if $V_{\beta }=\left\{f|f=\left(f^{0},f^{1},\cdots ,f^{N}\right)\right\}$ is the grid function space, then for any grid function $f\in V_{\beta }$, the FCFDS operator is defined as:*

(6) $$\begin{array}{c} {}_{0}D_{\beta }^{\alpha }f^{n}=\frac{1}{\beta^{\alpha }}[\rho_{1}\sum_{k=0}^{n}g_{k}^{\left(\alpha \right)}f^{n-k}+\rho_{2}\sum_{k=0}^{n-1}g_{k}^{\left(\alpha \right)}f^{n-k-1}+\\ \rho_{3}\sum_{k=0}^{n-2}g_{k}^{\left(\alpha \right)}f^{n-k-2}] \end{array}$$

*where n=2,3,⋯,N.*

(7) $$\begin{array}{c} \frac{\partial^{\alpha }f\left(x,y\right)}{\partial x^{\alpha }}=\rho_{1}f\left(x,y\right)+\left(-\alpha \rho_{1}+\rho_{2}\right)f\left(x-1,y\right)+\\ \left(\frac{\Gamma (2-\alpha )}{2!\Gamma (-\alpha )}\rho_{1}-\alpha \rho_{2}+\rho_{3}\right)f\left(x-2,y\right)+\\ \cdots +(\frac{\Gamma (N-\alpha )}{N!\Gamma (-\alpha )}\rho_{1}+\frac{\Gamma (N-\alpha -1)}{\left(N-1)!\Gamma (-\alpha \right)}\rho_{2}+\\ \frac{\Gamma (N-\alpha -2)}{\left(N-2)!\Gamma (-\alpha \right)}\rho_{3})f\left(x-N+1,y\right) \end{array}$$

(8) $$\begin{array}{c} \frac{\partial^{\alpha }f\left(x,y\right)}{\partial y^{\alpha }}=\rho_{1}f\left(x,y\right)+\left(-\alpha \rho_{1}+\rho_{2}\right)f\left(x,y-1\right)+\\ \left(\frac{\Gamma (2-\alpha )}{2!\Gamma (-\alpha )}\rho_{1}-\alpha \rho_{2}+\rho_{3}\right)f\left(x,y-2\right)+\\ \cdots +(\frac{\Gamma (N-\alpha )}{N!\Gamma (-\alpha )}\rho_{1}+\frac{\Gamma (N-\alpha -1)}{\left(N-1)!\Gamma (-\alpha \right)}\rho_{2}+\\ \frac{\Gamma (N-\alpha -2)}{\left(N-2)!\Gamma (-\alpha \right)}\rho_{3})f\left(x,y-N+1\right) \end{array}$$

From Equation (6), we can see that the FCFDS operator can be simplified to approximate expressions for multiplication and addition. When β=1t for a two-dimensional image f(x,y), the corresponding expressions in the horizontal *x* and vertical directions *y* directions are shown in Equations (7) and (8).

Since the structural information of the OCT images is concentrated in the horizontal and vertical directions, fractional differential masks are constructed in the directions of the positive *x*-coordinate and positive *y*-coordinate, respectively, as shown in Table 1 and Table 2. Note that $c_{k},k=1,2,\cdots ,n$ is the mask coefficient of the pixel of interest. When k←N=2s−2, the fractional differential mask (2s−1)×(2s−1) can be implemented. To make sure fractional differential mask has the certain center, in general, an even number is selected as *N*. It can be seen from Equations (7) and (8) that the mask coefficient $c_{k},k=1,2,\cdots ,n$ can be expressed as:

(9) $$\begin{array}{c} \left\{\begin{array}{c} c_{1}=\rho_{1}\\ c_{2}=-\alpha \rho_{1}+\rho_{2}\\ \vdots \\ c_{k}=\frac{\Gamma (k-\alpha )}{k!\Gamma (-\alpha )}\rho_{1}+\frac{\Gamma (k-\alpha -1)}{\left(k-1)!\Gamma (-\alpha \right)}\rho_{2}+\frac{\Gamma (k-\alpha -2)}{\left(k-2)!\Gamma (-\alpha \right)}\rho_{3}\\ \vdots \\ c_{N-1}=\frac{\Gamma (N-1-\alpha )}{\left(N-1)!\Gamma (-\alpha \right)}\rho_{1}+\frac{\Gamma (N-\alpha -2)}{\left(N-2)!\Gamma (-\alpha \right)}\rho_{2}+\frac{\Gamma (N-\alpha -3)}{\left(N-3)!\Gamma (-\alpha \right)}\rho_{3}\\ c_{N}=\frac{\Gamma (N-\alpha )}{N!\Gamma (-\alpha )}\rho_{1}+\frac{\Gamma (N-\alpha -1)}{\left(N-1)!\Gamma (-\alpha \right)}\rho_{2}+\frac{\Gamma (N-\alpha -2)}{\left(N-2)!\Gamma (-\alpha \right)}\rho_{3} \end{array}\right. \end{array}$$

For simplicity, let $\Delta_{x}^{\alpha }=\frac{\partial^{\alpha }f\left(x,y\right)}{\partial x^{\alpha }}$ and $\Delta_{y}^{\alpha }=\frac{\partial^{\alpha }f\left(x,y\right)}{\partial y^{\alpha }}$. The construction of the traditional mean operator leads to the loss of image details. To better preserve the details of the image, based on the FCFDS operator, we calculate a new similarity weighted kernel function. The new weight value can be defined as

(10) $$\omega \left(x,t\right)=e^{\frac{-\sum_{\Delta \in \Theta }ℜ\left(\Delta ,x\right)\left(v\left(x+\Delta \right)-v\left(t+\Delta \right)\right)}{h^{2}}}.$$

where v(•) is a pixel value at the position • from a noisy image *v*; w(x,t) is used to evaluate the similarity between the pixel intensity of two image blocks, where *x* and *t* represent two square image blocks of size k×k. As a filtering parameter, h controls the degree of decay of the exponential function. In addition, Δ can be represented as $\left(\Delta_{x},\Delta_{y}\right)$, where $(\Delta_{x},\Delta_{y})\in \Delta$ and Δ∈Θ; Θ can represent the image block region; ℜ(Δ,x) represents the kernel function.

Unlike the traditional convolutional Gaussian kernel function, the new kernel function ℜ(Δ,x) is constructed based on the FCFDS operator and contains more non-local information. ℜ(Δ,x) can be written as:

(11) $$ℜ\left(\Delta ,x\right)=e^{-(\frac{\sigma_{x}^{2}}{2\phi_{x}}+\frac{\sigma_{y}^{2}}{2\phi_{y}\left(\Delta ,x\right)})}$$

where $\sigma_{x}\in \left[-k,k\right]$ and $\sigma_{y}\in \left[-k,k\right]$; $\phi_{x}$ and $\phi_{y}\left(\Delta ,x\right)$ represent the variance parameters of the image block in the horizontal and vertical directions, respectively. Notably, the $\phi_{x}$ is a constant, and $\phi_{y}\left(\Delta ,x\right)$ is determined by the FCFDS operator, and its expression is:

(12) $$\phi_{y}\left(\Delta ,x\right)=\frac{1}{1+\frac{\Delta_{x}^{\alpha }+\Delta_{y}^{\alpha }}{\eta }}$$

where η is a damping coefficient, which determines the sensitivity of the $\phi_{y}\left(\Delta ,x\right)$ value depends on $\Delta_{x}^{\alpha }$ and $\Delta_{y}^{\alpha }$.

In this study, we use the weight function constructed by Equation (10) to obtain the filtered result image by weighted average, which is defined as

(13) $$\hat{v}\left(x\right)=\frac{\sum_{t\in F(x)}\omega \left(x,t\right)v\left(t\right)}{\sum_{t\in F(x)}\omega \left(x,t\right)}.$$

where F(x) is a set of neighborhood pixels around *x*, which are referred to as searching window.

To summarize, the pseudo-code of the proposed method is described in the Algorithm 1.
**Algorithm 1.** Proposed method for OCT image denoising.1:Input: Noisy image *v*;2:Initialize: The size of the search window *l*, the size of the similarity window *k*;3:Replicate the boundaries of the input image;4:Construct the FCFDS template operator through mask (1) and (2).5:Calculate the variance parameter $\phi_{y}\left(\Delta ,x\right)$ in the vertical direction by Equation (12);6:Calculate the kernel function ℜ(Δ,x) through Equation (11);7:Perform weight averaging by Equation (13);8:Output: Estimated image $\hat{v}$.

## 2. Experimental Results

**Figure 1:**
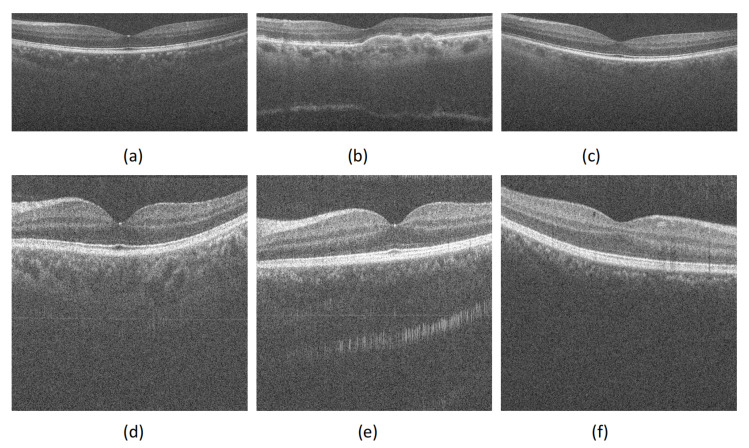
Representative images in each dataset. (**a**) DUKE-OCT1, (**b**) DUKE-OCT5, (**c**) DUKE-OCT7, (**d**) PKU37-OCT1, (**e**) PKU37-OCT2 and (**f**) PKU37-OCT9.

In order to verify the effectiveness of the algorithm, we perform noise removal experiments on real OCT retinal images, and some representative test images are shown in Figure 1. Where (a)–(c) and (d)–(f) in Figure 1 were provided online by the authors of Reference [15] and [38], respectively. Note that the DUKE dataset in Reference [15] and the PKU37 dataset in Reference [38] provide clean label images, that is, the average image of multiple adjacent images. In addition, for performing visual inspection and a quantitative comparison, the proposed algorithm was compared and analyzed against the outputs obtained after using NCDF [9], PNLM [12], WGLRR [19] and PBFD [3] four more advanced denoising algorithms.

The quantitative indicators used include Peak Signal-to-Noise Ratio (PSNR) [39] and Structural Similarity Index Measurement (SSIM) [39]. PSNR is mainly used to measure the sharpness, contrast and clarity of images. The larger their values, the better the structure of the image such as edges and textures is preserved. SSIM is mainly used to measure the smoothness of the image. The larger their value, the greater the smoothness of the image. All the algorithms are implemented using MATLAB programming on a laptop with the following hardware specifications: 3 GHz CPU and 16 GB RAM.

### 2.1. Visual Analysis of Real OCT Image Despeckling

**Figure 2:**
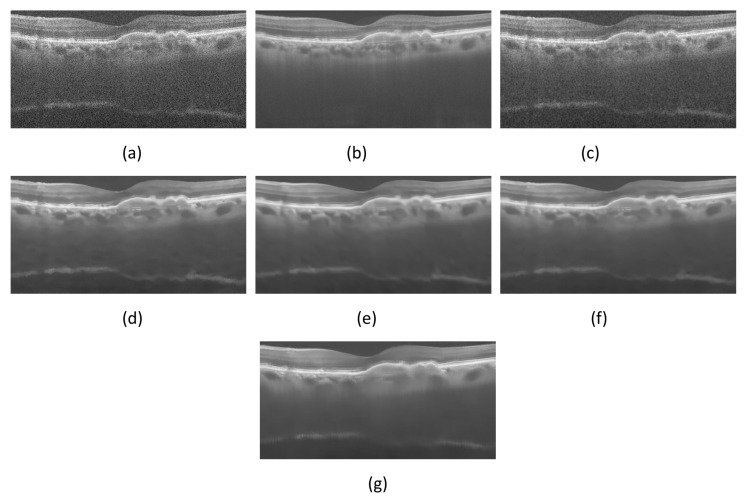
Comparison of despeckling effects of different despeckling algorithms on DUKE-OCT5 images. (**a**) Noisy image. (**b**) Clean image. (**c**–**g**) are the despeckled images obtained using the NCDF, PNLM, WGLRR, PBFD and Proposed method, respectively.

**Figure 3:**
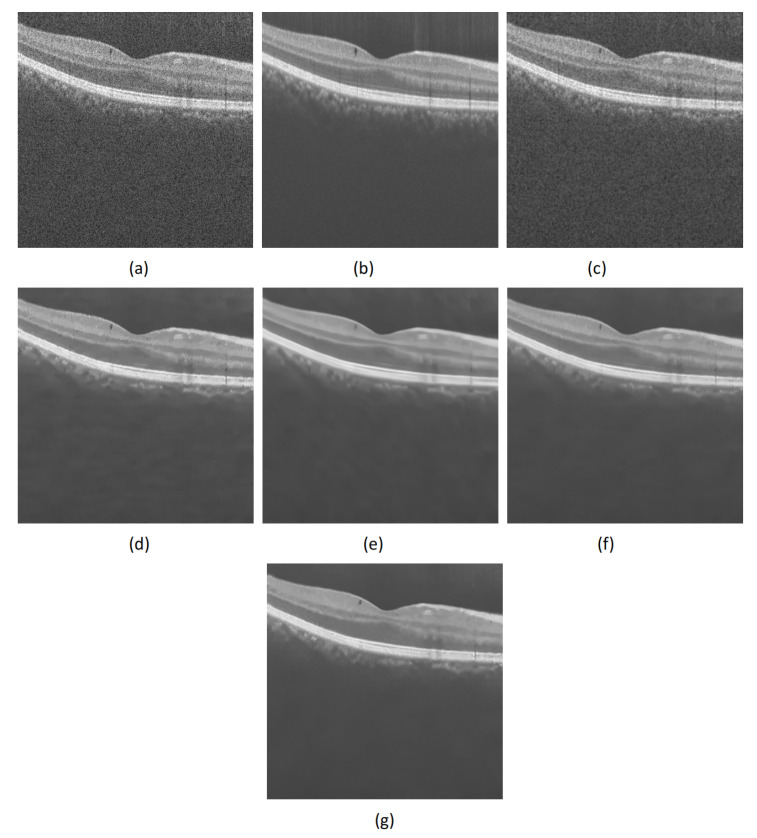
Comparison of despeckling effects of different despeckling algorithms on PKU37-OCT9 images. (**a**) Noisy image. (**b**) Clean image. (**c**–**g** ) are the despeckled images obtained using the NCDF, PNLM, WGLRR, PBFD and Proposed method, respectively.

The speckle noise removal experiment was carried out on the real OCT retinal test image, and the denoising effects of NCDF, PNLM, WGLRR and PBFD algorithms were compared. Some visual quality results after denoising are shown in Figure 2 and Figure 3.

From Figure 2 and Figure 3, it can be seen that the denoising result of NCDF algorithm is compromised. Although the NCDF algorithm reduces some speckle noise on OCT image, its structure information is still blurred. In addition, the PNLM algorithms can fully remove the noise in the background area, but the high-intensity structure area still contains some noise and the layer structure information is not clear. Furthermore, one finds that the WGLRR algorithm and the PBFD algorithm can fully remove speckle noise on OCT images, but over-smoothing may lead to layer structure confusion in the edge region of the filtered image. Finally, one sees that the filtering effect of the proposed algorithm is similar to that of the WGLRR and PBFD algorithms. It can not only reduce the speckle noise in the image, but also preserve the strong and weak edge structure information and detail information of the image. The estimated image obtained by the proposed method can well preserve the edges and features in the OCT image, which is very useful for other medical image analysis, such as edge detection, image layer segmentation and clinical diagnosis.

### 2.2. Quantitative Analysis of Real OCT Image Despeckling

In order to more clearly show the robustness and superiority of the proposed algorithm, in addition to the previous visual effect comparison analysis, we also quantitatively analyze the filtered images of the proposed algorithm and the four comparison algorithms, and the results are shown in Table 3 and Table 4. Note that since the clean label image given in the dataset is obtained by weighted average of multiple frames, there is a certain error in the calculation of PSNR and SSIM values, but we believe that the error is acceptable.

From Table 3, one finds that the PSNR values of the proposed algorithm for DUKE and PKU37 test images are greater than the PSNR values of the NCDF and PNLM algorithms, so the smoothing effect of the proposed algorithm is better than the NCDF and PNLM algorithms. In addition, one can also see that although the PSNR mean of the proposed algorithm is lower than the PSNR mean of the WGLRR and PBFD algorithms, he difference between their values is small and the PSNR value of the proposed algorithm for some test images in the DUKE dataset is better than these two algorithms. Therefore, the smoothing effect of the proposed algorithm for DUKE test images is similar to that of WGLRR and PBFD algorithms.

From Table 4, it can be seen that the SSIM values of the proposed algorithm for DUKE test images are similar to the results of PSNR. The SSIM values of the filtered images of the proposed algorithm are greater than those of NCDF and PNLM algorithms. The SSIM values of individual images have achieved the best results, and the SSIM values of some filtered images are greater than those of the PBFD algorithm. Therefore, for the Duke dataset, the ability of the proposed algorithm to preserve the structure is better than that of NCDF and PNLM algorithms, which is comparable to WGLRR and PBFD algorithms. In addition, for the PKU37 dataset, although the proposed algorithm does not obtain the highest SSIM values, their values are not the worst, that is, the proposed algorithm can not only better reduce speckle noise but also better preserve the structural information of the image.

In summary, the proposed methods for different test images are robust and have better denoising effect. Such an output image can effectively promote its subsequent application in scientific research.

### 2.3. Analysis of Main Contributions

**Figure 4:**
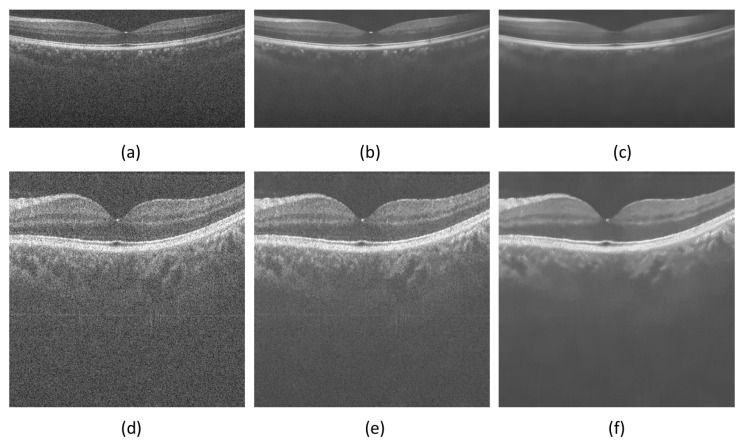
Comparison of OCT denoising using different kernel functions. (**a**) DUKE-OCT1, (**b**,**c**) respectively represents the filtering results of image (**a**) using traditional uniform kernel function and FCFDS kernel function. (**d**) PKU37-OCT1, (**e**,**f**) represents the filtering results of image (**d**) using traditional uniform kernel function and FCFDS kernel function, respectively.

**Figure 5:**
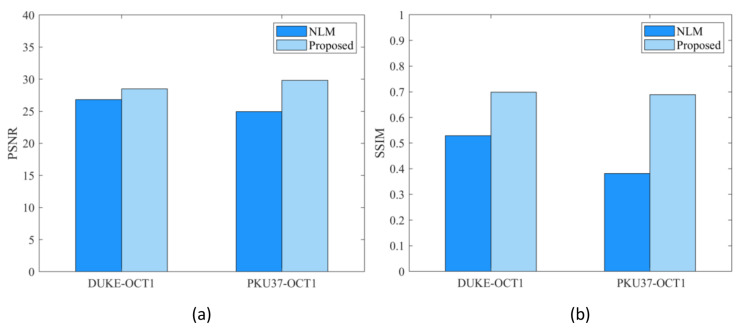
(**a**) and (**b**) represent the PSNR and SSIM results of the proposed method and the NLM algorithm for DUKE-OCT1 and PKU37-OCT1 test images, respectively.

The main contribution of this paper is to propose a new kernel function based on FCFDS format, and use the new kernel function to construct high-precision weighted operator. In order to better demonstrate the effectiveness of the proposed new kernel function, we used the same algorithm and parameters to conduct denoising experiments with the traditional kernel function and the new kernel function respectively. The experimental results are shown in Figure 4 and Figure 5.

It can be seen from Figure 4 that the new kernel function can greatly improve the filtering results of OCT images. The filtering result of the NLM algorithm using the traditional kernel function is under-smooth. The proposed algorithm using the new kernel function can effectively reduce the speckle noise of the OCT image and preserve its structural information. In addition, from Figure 5, one sees that the PSNR and SSIM results obtained by the algorithm using the new kernel function are much larger than the NLM algorithm using the traditional kernel function. Therefore, the validity of the new kernel function is demonstrated again from the perspective of quantitative index analysis.

### 2.4. Visual Analysis on OCT Data

**Figure 6:**
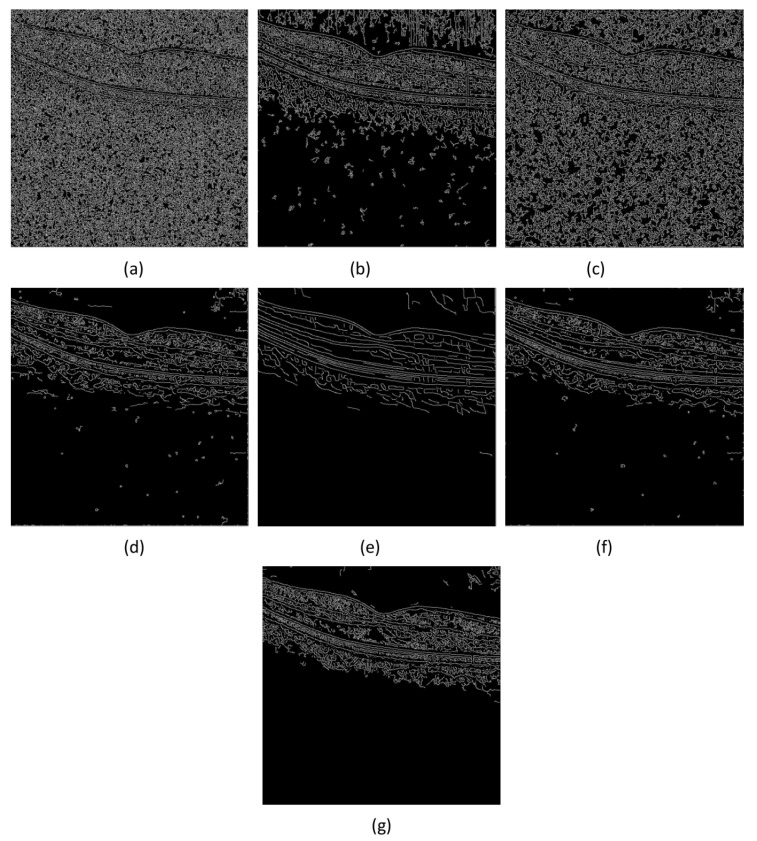
Comparison of edge detection results on denoised PKU37-OCT9 images in Figure 3. (**a**) Noisy image, (**b**) Clean image, results by (**c**) NCDF, (**d**) PNLM, (**e**) WGLRR, (**f**) PBFD and (**g**) Proposed method, respectively.

The OCT estimated image after speckle removal is an important tool for post-processing such as edge detection and segmentation. In order to further evaluate the effectiveness of the proposed algorithm, we use Canny edge detection algorithm [40] to test the edge of the denoised image. The results of image edge detection after denoising by each comparison method are shown in Figure 6.

From Figure 6, one finds that all comparison algorithms except the NCDF algorithm can better detect strong edge information on the image. However, the filtering result of WGLRR algorithm is too smooth, weak edges and details are difficult to detect. In addition, similar to clean label images, one also observes that the PNLM algorithm, FBFP algorithm and the proposed algorithm can all detect strong and weak edges and details, but it can be seen from Figure 6 that the noise in the background region of the PNLM algorithm and FBFP algorithm is also detected. In short, through edge detection, it can be found that the proposed algorithm has well removed the noise of the image, and clearly displayed the layer structure of the image, and the weak boundary layer has been well preserved.

## 3. Conclusions

We proposed an NLM algorithm based on the FCFDS to reduce the speckle noise in OCT images. The proposed algorithm primarily utilizes the non-local self-similarity, statistical properties, and FCFDS operator theory of the image itself. To examine the validity of the approach applied in this study, the experimental results were compared with those published in the previous research, and a good agreement was observed between them. The experiments results revealed that:

The bidirectional high-precision image similarity weighting strategy based on the FCFDS is more effective than the traditional isotropic Gaussian similarity weighting strategy for denoising the OCT images.

Compared to some commonly used methods to remove speckle noise, our proposed method obtains the better results in both visual inspection and quantitative index analysis.

Therefore, the denoising algorithm proposed herein is expected to be applied in a wide range of biomedical imaging applications and to promote scientific research related to its subsequent processing.

## Figures and Tables

**Table 1:** Fractional differential mask on the directions of positive *x*-coordinate.

	⋯	0	$c_{1}$	0	⋯	
	⋯	0	$c_{2}$	0	⋯	
	⋯	0	⋮	0	⋯	
	⋯	0	$c_{k}$	0	⋯	
	⋯	0	⋮	0	⋯	
	⋯	0	$c_{N-1}$	0	⋯	
	⋯	0	$c_{N}$	0	⋯	

**Table 2:** Fractional differential mask on the directions of positive *y*-coordinate.

						
⋮	⋮	⋮	⋮	⋮	⋮	⋮
0	0	⋯	⋯	0	0	0
$c_{1}$	$c_{2}$	⋯	$c_{k}$	⋯	$c_{N-1}$	$c_{N}$
0	0	⋯	⋯	0	0	0
⋮	⋮	⋮	⋮	⋮	⋮	⋮
						

**Table 3:** The PSNR results of five different algorithms for DUKE and PKU37 test images.

PSNR	Noisy	NCDF	WGLRR	PNLM	PBFD	Proposed
DUKE-OCT1	27.9308	25.4006	28.5339	27.6734	28.4816	28.5084
DUKE-OCT2	27.7130	26.3255	30.4971	29.3200	30.5217	30.2391
DUKE-OCT3	27.9057	25.7065	28.5744	27.9491	28.3597	28.2461
DUKE-OCT4	27.8436	27.0619	30.3636	29.9466	30.4783	30.0756
DUKE-OCT5	28.1309	24.9686	28.1948	27.3035	27.9387	28.1336
DUKE-OCT6	27.5377	25.4821	28.3020	27.5794	28.3315	28.3665
DUKE-OCT7	27.9631	26.3100	30.4909	29.3305	30.3201	30.2102
DUKE-OCT8	28.0687	26.4289	30.0981	29.5000	29.8778	29.6921
DUKE-OCT9	27.7863	24.1477	25.9196	25.5260	26.0550	26.0007
DUKE-OCT10	27.6330	26.9981	31.2946	30.2993	31.1520	30.9526
Mean	27.8513	25.8830	29.2269	28.4428	29.1516	29.0425
Standard Deviation	0.1876	0.9178	1.6091	1.4821	1.5755	1.4677
PKU37-OCT1	27.7825	26.2985	31.1508	29.6228	31.3268	29.8319
PKU37-OCT2	27.7166	25.8411	29.8476	28.4225	30.1044	29.8319
PKU37-OCT3	27.7165	26.4996	31.3394	29.3470	31.3170	30.4102
PKU37-OCT4	28.2921	28.2967	31.7332	30.8740	31.8101	31.0674
PKU37-OCT5	28.1621	27.8008	30.4687	29.7409	30.5977	30.0595
PKU37-OCT6	28.2306	28.6672	32.1720	31.4908	32.3373	31.8362
PKU37-OCT7	28.1526	27.5770	31.5276	30.4475	31.5662	31.0027
PKU37-OCT8	27.8084	27.4455	29.7897	28.9533	30.1912	29.8630
PKU37-OCT9	28.3918	27.1661	31.2882	30.5118	31.2136	30.5134
PKU37-OCT10	28.1823	27.9849	31.5513	30.7195	31.5836	30.3887
Mean	28.0436	27.3577	31.0869	30.0130	31.2048	30.4805
Standard Deviation	0.2583	0.9090	0.7968	0.9536	0.7116	0.6557

**Table 4:** The SSIM results of five different algorithms for DUKE and PKU37 test images.

SSIM	Noisy	NCDF	WGLRR	PNLM	PBFD	Proposed
DUKE-OCT1	0.0810	0.4763	0.6990	0.6622	0.6937	0.6991
DUKE-OCT2	0.0855	0.4807	0.7111	0.6686	0.7018	0.7051
DUKE-OCT3	0.0827	0.4814	0.6962	0.6636	0.6805	0.6940
DUKE-OCT4	0.1295	0.5347	0.7026	0.6959	0.7061	0.7006
DUKE-OCT5	0.0817	0.4614	0.7018	0.6314	0.6858	0.6907
DUKE-OCT6	0.0787	0.4714	0.6959	0.6610	0.6791	0.6924
DUKE-OCT7	0.0852	0.4884	0.7223	0.6751	0.7116	0.7142
DUKE-OCT8	0.0926	0.4997	0.7176	0.6912	0.7055	0.7134
DUKE-OCT9	0.0645	0.4393	0.6511	0.6096	0.6472	0.6572
DUKE-OCT10	0.0847	0.4945	0.7227	0.6919	0.7082	0.7164
Mean	0.0866	0.4828	0.7020	0.6651	0.6919	0.6983
Standard Deviation	0.0167	0.0251	0.0206	0.0273	0.0196	0.0172
PKU37-OCT1	0.1236	0.5118	0.7211	0.7248	0.7309	0.6887
PKU37-OCT2	0.1357	0.5100	0.6939	0.7029	0.7097	0.6887
PKU37-OCT3	0.1085	0.5029	0.7147	0.7097	0.7169	0.6876
PKU37-OCT4	0.1724	0.6431	0.7912	0.7969	0.8017	0.7869
PKU37-OCT5	0.1764	0.6123	0.7345	0.7448	0.7479	0.7353
PKU37-OCT6	0.1634	0.6303	0.7734	0.7814	0.7849	0.7743
PKU37-OCT7	0.1387	0.6081	0.8005	0.7984	0.8064	0.7902
PKU37-OCT8	0.1945	0.6222	0.7405	0.7596	0.7621	0.7528
PKU37-OCT9	0.1746	0.5948	0.7583	0.7594	0.7668	0.7547
PKU37-OCT10	0.1729	0.5982	0.7366	0.7417	0.7464	0.7260
Mean	0.1561	0.5834	0.7465	0.7520	0.7574	0.7385
Standard Deviation	0.0276	0.0538	0.0341	0.0337	0.0334	0.0401

## Data Availability

Data underlying the results presented in this paper are not publicly available at this time but may be obtained from the authors upon reasonable request.

## References

[1] Huang D., Swanson E.A., Lin C.P., Schuman J.S., Stinson W.G., Chang W., Hee M.R., Flotte T., Gregory K., Puliafito C.A. (1991). Optical coherence tomography. Science.

[2] Schmitt J.M., Xiang S., Yung K.M. (1999). Speckle in optical coherence tomography. J. Biomed. Opt..

[3] Chen H. (2021). Fusion denoising algorithm of optical coherence tomography image based on point-estimated and block-estimated. Optik.

[4] Zhang P., Miller E.B., Manna S.K., Meleppat R.K., Pugh E.N., Zawadzki R. (2019). Temporal speckle-averaging of optical coherence tomography volumes for in-vivo cellular resolution neuronal and vascular retinal imaging. Neurophotonics.

[5] Zhang P., Manna S.K., Miller E.B., Jian Y., Meleppat R.K., Sarunic M.V., Pugh E.N., Zawadzki R.J. (2019). Aperture phase modulation with adaptive optics: A novel approach for speckle reduction and structure extraction in optical coherence tomography. Biomed. Opt. Express.

[6] Adler D.C., Ko T.H., Fujimoto J.G. (2004). Speckle reduction in optical coherence tomography images by use of a spatially adaptive wavelet filter. Opt. Lett..

[7] Zaki F., Wang Y., Su H., Yuan X., Liu X. (2017). Noise adaptive wavelet thresholding for speckle noise removal in optical coherence tomography. Biomed. Qptics Express.

[8] Jian Z., Yu Z., Yu L., Rao B., Chen Z., Tromberg B.J. (2009). Speckle attenuation in optical coherence tomography by curvelet shrinkage. Opt. Lett..

[9] Bernardes R., Maduro C., Serranho P., Araújo A., Barbeiro S., Cunha-Vaz J. (2010). Improved adaptive complex diffusion despeckling filter. Opt. Express.

[10] Duan J., Lu W., Tench C., Gottlob I., Proudlock F., Samani N.N., Bai L. (2016). Denoising optical coherence tomography using second order total generalized variation decomposition. Biomed. Signal Process. Control.

[11] Aum J., Kim J.h., Jeong J. (2015). Effective speckle noise suppression in optical coherence tomography images using nonlocal means denoising filter with double Gaussian anisotropic kernels. Appl. Opt..

[12] Yu H., Gao J., Li A. (2016). Probability-based non-local means filter for speckle noise suppression in optical coherence tomography images. Opt. Lett..

[13] Zhou Q., Guo J.M., Ding M., Zhang X. (2020). Guided Filtering based Nonlocal Means Despeckling of Optical Coherence Tomography Images. Opt. Lett..

[14] Fang L., Li S., Nie Q., Izatt J.A., Toth C.A., Farsiu S. (2012). Sparsity based denoising of spectral domain optical coherence tomography images. Biomed. Opt. Express.

[15] Fang L., Li S., McNabb R.P., Nie Q., Kuo A.N., Toth C.A., Izatt J.A., Farsiu S. (2013). Fast acquisition and reconstruction of optical coherence tomography images via sparse representation. IEEE Trans. Med. Imaging.

[16] Huang S., Tang C., Xu M., Qiu Y., Lei Z. (2019). BM3D-based total variation algorithm for speckle removal with structure-preserving in OCT images. Appl. Opt..

[17] Zhang X., Li Z., Nan N., Wang X. (2022). Denoising algorithm of OCT images via sparse representation based on noise estimation and global dictionary. Opt. Express.

[18] Tang C., Zheng X., Cao L. (2017). OCT despeckling via weighted nuclear norm constrained non-local low-rank representation. Laser Phys. Lett..

[19] Tang C., Cao L., Chen J., Zheng X. (2017). Speckle noise reduction for optical coherence tomography images via non-local weighted group low-rank representation. Laser Phys. Lett..

[20] Chen H., Fu S., Wang H., Lv H., Zhang C., Wang F., Li Y. (2019). Feature-oriented singular value shrinkage for optical coherence tomography image. Opt. Lasers Eng..

[21] Mayer M.A., Borsdorf A., Wagner M., Hornegger J., Mardin C.Y., Tornow R.P. (2012). Wavelet denoising of multiframe optical coherence tomography data. Biomed. Opt. Express.

[22] Thapa D., Raahemifar K., Lakshminarayanan V. (2015). Reduction of speckle noise from optical coherence tomography images using multi-frame weighted nuclear norm minimization method. J. Mod. Opt..

[23] Lv H., Fu S., Zhang C., Zhai L. (2018). Speckle noise reduction of multi-frame optical coherence tomography data using multi-linear principal component analysis. Opt. Express.

[24] Wang C., You Y.J., Ai S., Zhang W., Liao W., Zhang X., Hsieh J., Zhang N., Tang B., Pan C.L. (2019). Multi-frame speckle reduction in OCT using supercontinuum pumped by noise-like pulses. J. Innov. Opt. Health Sci..

[25] Huang Y., Lu Z., Shao Z., Ran M., Zhou J., Fang L., Zhang Y. (2019). Simultaneous denoising and super-resolution of optical coherence tomography images based on generative adversarial network. Opt. Express.

[26] Gour N., Khanna P. (2020). Speckle denoising in optical coherence tomography images using residual deep convolutional neural network. Multimed. Tools Appl..

[27] Xu M., Tang C., Chen M., Qiu Y., Lei Z. (2019). Texture preservation and speckle reduction in optical coherence tomography using the shearlet-based total variation algorithm. Opt. Lasers Eng..

[28] Chen Z., Zeng Z., Shen H., Zheng X., Dai P., Ouyang P. (2020). DN-GAN: Denoising generative adversarial networks for speckle noise reduction in optical coherence tomography images. Biomed. Signal Process. Control.

[29] Coupé P., Hellier P., Kervrann C., Barillot C. (2009). Nonlocal means-based speckle filtering for ultrasound images. IEEE Trans. Image Process..

[30] Mei F., Zhang D., Yang Y. (2020). Improved non-local self-similarity measures for effective speckle noise reduction in ultrasound images. Comput. Methods Programs Biomed..

[31] Torres L., Sant’Anna S.J., da Costa Freitas C., Frery A.C. (2014). Speckle reduction in polarimetric SAR imagery with stochastic distances and nonlocal means. Pattern Recognit..

[32] Cuartas-Vélez C., Restrepo R., Bouma B.E., Uribe-Patarroyo N. (2018). Volumetric non-local-means based speckle reduction for optical coherence tomography. Biomed. Opt. Express.

[33] Chen H., Fu S., Wang H., Li Y., Wang F. (2019). Speckle reduction based on fractional-order filtering and boosted singular value shrinkage for optical coherence tomography image. Biomed. Signal Process. Control.

[34] Kilbas A.A., Srivastava H.M., Trujillo J.J. (2006). Theory and Applications of Fractional Differential Equations.

[35] Meerschaert M.M., Tadjeran C. (2004). Finite difference approximations for fractional advection–dispersion flow equations. J. Comput. Appl. Math..

[36] Tian W., Zhou H., Deng W. (2015). A class of second order difference approximations for solving space fractional diffusion equations. Math. Comput..

[37] Ji C.c., Sun Z.z. (2015). A high-order compact finite difference scheme for the fractional sub-diffusion equation. J. Sci. Comput..

[38] Geng M., Meng X., Zhu L., Jiang Z., Gao M., Huang Z., Qiu B., Hu Y., Zhang Y., Ren Q. (2022). Triplet Cross-Fusion Learning for Unpaired Image Denoising in Optical Coherence Tomography. IEEE Trans. Med. Imaging.

[39] Hore A., Ziou D. Image quality metrics: PSNR vs. SSIM. Proceedings of the 2010 20th International Conference on Pattern Recognition.

[40] Canny J. (1986). A computational approach to edge detection. IEEE Trans. Pattern Anal. Mach. Intell..

